# The Potency of Seaweed Sulfated Polysaccharides for the Correction of Hemostasis Disorders in COVID-19

**DOI:** 10.3390/molecules26092618

**Published:** 2021-04-29

**Authors:** Tatyana A. Kuznetsova, Boris G. Andryukov, Ilona D. Makarenkova, Tatyana S. Zaporozhets, Natalya N. Besednova, Ludmila N. Fedyanina, Sergey P. Kryzhanovsky, Mikhail Yu. Shchelkanov

**Affiliations:** 1 G.P. Somov Institute of Epidemiology and Microbiology, Russian Federal Service for Surveillance on Consumer Rights Protection and Human Wellbeing, 690087 Vladivostok, Russia; andrukov_bg@mail.ru (B.G.A.); ilona_m@mail.ru (I.D.M.); niiem_vl@mail.ru (T.S.Z.); besednoff_lev@mail.ru (N.N.B.); adorob@mail.ru (M.Y.S.); 2 School of Biomedicine, Far Eastern Federal University (FEFU), 690091 Vladivostok, Russia; fedyanina.ln@dvfu.ru; 3 Medical Association of the Far Eastern Branch of the Russian Academy of Sciences, 690022 Vladivostok, Russia; priemmodvoran@mail.ru; 4 Federal Scientific Center of the Eastern Asia Terrestrial Biodiversity, Far Eastern Branch of Russian Academy of Sciences, 690091 Vladivostok, Russia; 5 National Scientific Center of Marine Biology, Far Eastern Branch of Russian Academy of Sciences, 690091 Vladivostok, Russia

**Keywords:** seaweed polysaccharides, fucoidans, carrageenans, ulvans, anticoagulants, thrombolytics, fibrinolytics, thrombin inhibitors, hemostasis disorders, blood coagulation, COVID-19 associated coagulopathy, SARS-CoV-2

## Abstract

Hemostasis disorders play an important role in the pathogenesis, clinical manifestations, and outcome of COVID-19. First of all, the hemostasis system suffers due to a complicated and severe course of COVID-19. A significant number of COVID-19 patients develop signs of hypercoagulability, thrombocytopenia, and hyperfibrinolysis. Patients with severe COVID-19 have a tendency toward thrombotic complications in the venous and arterial systems, which is the leading cause of death in this disease. Despite the success achieved in the treatment of SARS-CoV-2, the search for new effective anticoagulants, thrombolytics, and fibrinolytics, as well as their optimal dose strategies, continues to be relevant. The wide therapeutic potential of seaweed sulfated polysaccharides (PSs), including anticoagulant, thrombolytic, and fibrinolytic activities, opens up new possibilities for their study in experimental and clinical trials. These natural compounds can be important complementary drugs for the recovery from hemostasis disorders due to their natural origin, safety, and low cost compared to synthetic drugs. In this review, the authors analyze possible pathophysiological mechanisms involved in the hemostasis disorders observed in the pathological progression of COVID-19, and also focus the attention of researchers on seaweed PSs as potential drugs aimed to correction these disorders in COVID-19 patients. Modern literature data on the anticoagulant, antithrombotic, and fibrinolytic activities of seaweed PSs are presented, depending on their structural features (content and position of sulfate groups on the main chain of PSs, molecular weight, monosaccharide composition and type of glycosidic bonds, the degree of PS chain branching, etc.). The mechanisms of PS action on the hemostasis system and the issues of oral bioavailability of PSs, important for their clinical use as oral anticoagulant and antithrombotic agents, are considered. The combination of the anticoagulant, thrombolytic, and fibrinolytic properties, along with low toxicity and relative cheapness of production, open up prospects for the clinical use of PSs as alternative sources of new anticoagulant and antithrombotic compounds. However, further investigation and clinical trials are needed to confirm their efficacy.

## 1. Introduction

Hemostasis disorders play an important role in the pathogenesis, clinical manifestations, and outcome of COVID-19. First of all, the hemostasis system suffers due to a complicated and severe course of COVID-19. In the initial stage of the disease, a significant number of COVID-19 patients develop signs of hypercoagulation, and in the later stages, signs of disseminated intravascular coagulation (DIC) syndrome. Coagulation disorders and their clinical manifestations have a number of differences from the classic DIC syndrome, and are named COVID-associated coagulopathy [1,2,3].

Patients with severe COVID-19 have a tendency toward thrombotic complications in the venous and arterial systems, which is the leading cause of death in this disease [1,4,5,6].

The treatment of COVID-19 patients and, in particular, correction of the hemostasis disorders is a difficult problem. Despite the data on the effectiveness of heparin drugs in reducing mortality in severe COVID-19 patients, and despite thromboprophylaxis [7,8], the development of venous and arterial thromboembolic complications has been reported. There is also a problem with the optimal dosage of anticoagulant agent [5,9,10,11]. Thus, there are a number of unresolved issues regarding the use of these drugs.

In this regard, there is a need to use the effective anticoagulants and antithrombotic drugs, as well as their optimal dosages, in the treatment of COVID-19 patients and for the prevention of thrombotic complications [7,12,13].

Despite the success achieved in SARS-CoV-2 therapy, the development of more effective treatment regimens for this infection with the inclusion of anticoagulants, thrombolytics, and fibrinolytics, as well as the search for new effective drugs of this pharmacological group, does not lose relevance. The wide therapeutic potential of seaweed sulfated polysaccharides (PSs), including anticoagulant, thrombolytic, and fibrinolytic activities, opens up a new possibilities for their study in experimental and clinical trials.

This review presents current literature data on the anticoagulant, antithrombotic, and fibrinolytic activities of seaweed PSs, depending on their structural features (content and position of sulfate groups on the main chain of PS, molecular weight, monosaccharide composition and type of glycosidic bonds, the degree of PS chain branching, etc.). The mechanisms of PS action on the hemostasis system are discussed compared to heparin. The issues of oral bioavailability of PSs, important for their clinical use as oral anticoagulant and antithrombotic agents, are considered. The combination of the anticoagulant, thrombolytic, and fibrinolytic properties, along with low toxicity and the relative low cost of their production, opens up prospects for the clinical use of PSs as alternative sources of new anticoagulant and antithrombotic compounds, including those aimed to correction hemostasis disorders in COVID-19 patients.

## 2. Anticoagulant, Antithrombotic, and Fibrinolytic Activities of Seaweed Sulfated Polysaccharides

Polysaccharides (PSs) are the main structural components of brown, red, and green seaweed, the content of which can be up to 76% of the dry weight [14]. Seaweed sulfated PSs possess a wide spectrum of biological activity, including immunoregulatory, antitumor, anti-inflammatory, antiviral and antibacterial, antioxidant, anticoagulant, etc. This excellent biological activity is attributed to their unique biological structure.

The anticoagulant properties of seaweed sulfated PSs are widely described in the scientific literature. Springer et al. [15] were the first to detect anticoagulant activity in sulfated PS (fucoidan) from the brown alga *Fucus vesiculosus*. Further numerous studies have confirmed that seaweed sulfated PSs are strong anticoagulants and may be alternative sources of new anticoagulant compounds.

**Figure 1:**
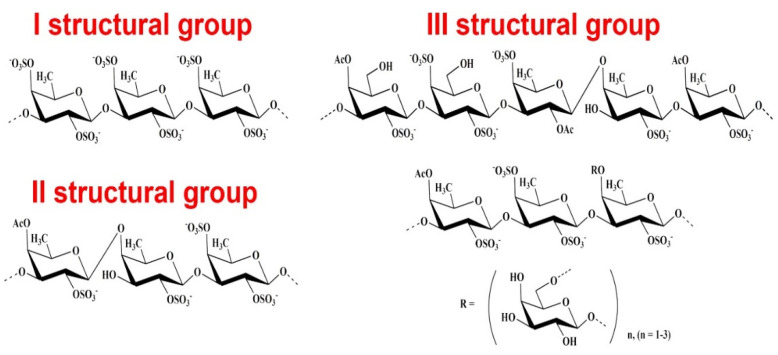
Structural fragments of fucoidans. Most of the known fucoidans belong to three structural types: the first type contains (1→3)-linked *L*-fucopyranose residues in the main chain; the second type is alternating (1→3)- and (1→4)-linked residues of L-fucopyranose; the third type of fucoidans (galactofucans) contains fucose and galactose residues, and sometimes these monosaccharides are represented in the structures of fucoidans in comparable amounts. In addition to fucose, fucoidans often contain small amounts of other monosaccharides.

The study of seaweed sulfated PS anticoagulant activity and its mechanisms is carried out mainly in connection with the structural features of these compounds. So, sulfated PSs of brown algae (fucoidans) represent the family of fucose-containing homo- and heteropolysaccharides from polysaccharides with a high content of uronic acids and low fucose and sulfate contents for practically pure α-L-fucans with the main component of polysaccharide-fucose. Except for fucose, these polysaccharides can contain minor amounts of other monosaccharides (galactose, mannose, xylose, glucose) and also sulfates, uronic acids, acetyl groups, and proteins [16,17,18] (Figure 1).

**Figure 2:**
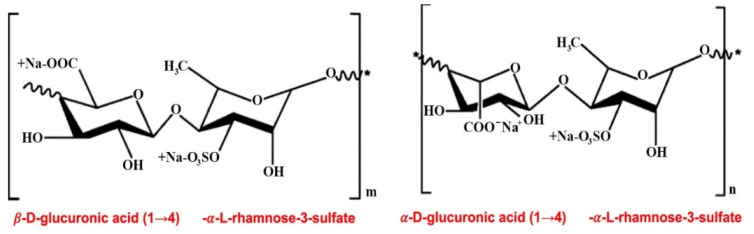
Structures of the main repeating disaccharides constituting ulvans. The backbone of the chemical structure of ulvans is composed of glucuronic acid and repeating disaccharide units (xylose and rhamnose): α- and β-(1→4)-linked sugar residues (such as α-1,4- and α-1,2,4-linked *L*-rhamnose), β-1,4- and terminally linked d-glucuronic acid, and β-1,4-linked d-xylose.

The backbone of the chemical structure of green algae sulfated PSs (ulvans) is composed of glucuronic acid and repeating disaccharide units (xylose and rhamnose): α- and β-(1→4)-linked sugar residues (such as α-1,4- and α-1,2,4-linked *L*-rhamnose), β-1,4- and terminally linked d-glucuronic acid, and β-1,4-linked d-xylose [19,20,21,22,23] (Figure 2).

**Figure 3:**
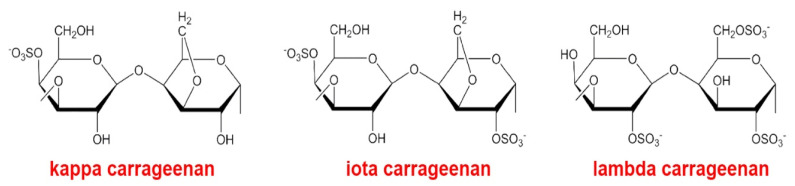
Structural fragments of carrageenans. Carrageenans are sulfated linear PSs whose chemical structure is based on a repeating disaccharide unit consisting of D-galactose residues connected via alternating β-1→4- and -1→3-glycosidic bonds. Sulfated PSs of red algae of the class Rhodophyceae consist of repeating dimers of α-1,4- D-galactose, which are connected via alternating α-1→3- and β-1→4-glycosidic bonds and substituted by one (κ-carrageenan), two (ι-carrageenan), or three (λ-carrageenan) sulfate ester groups in each repeating unit.

Carrageenans are sulfated linear PSs of red algae, whose chemical structure is based on a repeating disaccharide unit consisting of D-galactose residues connected via alternating β-1→4- and -1→3-glycosidic bonds. Thus, sulfated PSs of red algae of the class Rhodophyceae consist of repeating dimers of α-1,4- D-galactose, which are connected via alternating α-1→3- and β-1→4-glycosidic bonds and substituted by one (κ-carrageenan), two (ι-carrageenan), or three (λ-carrageenan) sulfate ester groups in each repeating unit [24,25,26] (Figure 3).

The relationship between anticoagulant activities of PSs and their chemical structure is complex. A number of factors, such as molecular weight (MW), monosaccharide composition, and content of sulfates, as well as the nature of the bonds in the polysaccharide chain, have a significant influence on the anticoagulant activity. At the same time, according to many researchers, the degree of anticoagulant action of sulfated PSs is closely correlated with their structure and function, and varies significantly from weakly expressed or absent to high.

First of all, the content of sulfates has the most significant effect on the anticoagulant potential of these PSs. A number of authors confirmed this relationship in well-studied fucoidans from brown algae with different content of sulfates, and showed that desulfated fractions of fucoidans were inactive in coagulological tests [27,28,29,30]. The results obtained by these authors also indicate that the PS fractions from different types and species of brown algae, enriched with sulfates, but poor with uronic acids, exhibit relatively high activity, while the fractions with the inverse ratio of these structural components have a weaker anticoagulant activity. As for PSs from different types of green algae, it also was demonstrated that high-molecular-weight sulfated galactans with a high content of sulfates have a higher anticoagulant activity than low-molecular-weight PSs with a low content of sulfates [31,32,33].

Thus, the authors of one of the recent works reported that the PSs obtained by additional sulfation fraction from *Ulva rigida*, called ULVAN-02 (while the content of sulfates increased to 20% compared to the ULVAN-01 fraction containing 11% of sulfates) showed a significant increase in anticoagulant activity regardless of the coagulation pathway [33]. This activity was comparable to that of heparin, and was higher than that of the commercial anticoagulant drug Lovenox^®^ (Sanofi-Aventis, Rock Hill, SC, USA). According to the authors, the most probable hypothesis explaining the anticoagulant activity of the ULVAN-02 fraction is that the geometric correspondence between PSs and their binding sites with coagulation factors increase with increasing degree of sulfation. At the same time, hypersulfated zones provide a higher physical ability to interact, which further increases the degree of PS binding. Taking into account the presented data, it can be assumed that the potential anticoagulants are PSs with a high degree of sulfation.

However, as noted by other authors, PSs from various green algae species with a high content of sulfates (26–35%) showed no activity with respect to the external coagulation pathway [34,35]. According to Silva et al. [29] and Yoon et al. [36], the anticoagulant activity of fucoidans from various algae species did not correlate with the content of sulfate groups, but were associated with the position of sulfates. It follows from these data that the degree of sulfation is not the only parameter that affects the anticoagulant activity of PSs. The monosaccharide composition and type of glycosidic bonds, the degree of chain branching, and the position of the sulfate groups on the main chain of the PSs are important too. This dependency is also very ambiguous. As is known, the main structural monosaccharides in the composition of PSs are fucose, galactose, mannose, xylose, and rhamnose in different ratios. Thus, it was found that a high content of fucose (as well as a sulfate residue) and a low content of other neutral sugars can contribute to the manifestation of high anticoagulant activity of fucoidans [29,36]. Therefore, the ratio of sulfates/total residual sugars has a certain value for the manifestation of anticoagulant properties (even to a greater extent than the degree of sulfation).

An important factor influencing the anticoagulant activity of PSs is the MW. According to Yang et al. [37], fucoidans exhibit the strongest anticoagulant activity in the range of MW from 10 to 300 kDa. Pomin et al. [38] confirmed the relationship between MW and anticoagulant activity and showed that a decrease in MW of fucoidan from *Laminaria brasiliensis* led to a sharp decrease in the latter. Boisson-Vidal et al. [39] also noted that the anticoagulant and antithrombotic activity of fucoidan fractions from *Ascophyllum nodosum* increased with increasing MW and sulfate content. The fucoidan fractions in which the natural structure was not degraded (i.e., the sulfate group was intact) were more active in the manifestation of the anticoagulant action than fractions with an equivalent MW and degree of sulfation, but modified by partial desulfation. Lahrsen et al. [40] also showed that the anticoagulant activity of fucoidan from *F. vesiculosus* and 18 gradually depolymerized fractions decreased with decreasing MW.

Therefore, some researchers believe that the anticoagulant activity of PSs is associated with a high MW (up to some hundred kDa), while others consider the lower limit of this activity to be 10 kDa, but in general, the authors concluded that different fucoidan fractions have their own optimal MW for the manifestation of such activity.

Numerous data indicate the antithrombotic activity of seaweed sulfated PSs. Thus, the studies of Choi et al. [41] demonstrated the thrombolytic activity of various fucoidan fractions from the brown algae *Fucus evanescens* and *Undaria pinnatifida*. The authors associated this activity with an increase in the level of t-PA (tissue plasminogen activator). The fucoidans inhibit the tPA-PAI-1 (plasminogen activator inhibitor-1) complex, indicating activation of plasma tissue-type plasminogen activator is a mechanism of fucoidan-mediated thrombolysis in a mouse thrombosis model [41]. Min et al. [42] also showed thrombolytic effects of fucoidans from different types of algae in a mouse arterial thrombosis model. Fucoidan infusion to mice resulted in a several-fold increase in t-PA levels compared to the control group (healthy animals) and the group of untreated animals with thrombosis (*p* < 0.01). The authors concluded that the mechanism of fucoidan-mediated thrombolysis is realized through the release of vascular endothelial t-PA, depending on the dose and type of algae [42]. Sulfated PSs from green alga *Monostroma nitidum* showed significant anticoagulant and antithrombotic properties in vitro and in vivo. The authors considered that this PS may be a promising drug for the prevention and treatment of thromboembolic diseases [43]. Juenet et al. [44] also investigated the thrombolytic effect of fucoidan in a mouse venous thrombosis model by monitoring the platelet density in the thrombus with intravital microscopy. Polysaccharide-poly(isobutylcyanoacrylate) nanoparticles functionalized with fucoidan (Algues & Mer, Ouessant, France) and loaded with rt-PA (recombinant tissue plasminogen activator) were designed to accumulate on the thrombus. These nanoparticles showed the greatest thrombolytic effect. The platelet density was decreased to 30%, whereas after the introduction of nanoparticles without fucoidan, the value of this parameter was 66%. The authors believe that the nanoparticles not only protect rt-PA, but also enhance its thrombolytic activity due to the ability of fucoidan-functionalized polymer nanoparticles targeting P-selectin [44]. This work provides the evidence for the concept of fucoidan-based carriers for targeted thrombolysis.

In addition, the fibrinolytic activity of fucoidans were shown. The mechanisms of fibrinolytic activity and activation of the endogenous fibrinolytic system are associated with an increase in the potential activity of the plasmin system [32,41,45,46]. Thus, Liu et al. [32] assessed the fibrinolytic and thrombolytic activity of sulfated PSs from the green alga *Monostroma angicava* and its low-molecular-weight fragments using in vitro and in vivo experiments (by D-dimer, fibrin degradation products, PAI-1, and clot lytic rate assays). The results showed that PSs with MW of 24–240 kDa possessed high fibrinolytic and thrombolytic activities [32].

As a result, the presented data of numerous studies indicate that the anticoagulant, fibrinolytic, and thrombolytic activity critically depends on some structural features. The features are the content and position of sulfate groups on the main chain of PSs, MW, monosaccharide composition and type of glycosidic bonds, the degree of PS chain branching, etc., but the degree of sulfation and monosaccharide composition are most important.

As for the mechanisms of anticoagulant activity of PSs, the study is usually carried out in a comparative aspect to heparin, the most widely used anticoagulant in medicine. However, the clinical use of heparin causes a number of side effects, including hemorrhagic effects and the occurrence of heparin-induced thrombocytopenia, thrombosis, hyperkalemia, osteopenia, etc. [47,48,49]. In addition, the therapeutic use of heparin is limited by parenteral administration (mainly due to the negative charge and large MW), in contrast to the new oral anticoagulants [50].

In this respect, low-toxic seaweed sulfated PSs represent an alternative to heparin.

The mechanisms of anticoagulant action of sulfated PSs from seaweed and heparin have a number of differences, as evidenced by the results of a number of studies. An important role in the implementation of the mechanisms of anticoagulant activity of sulfated PS, as well as heparin, belongs to thrombin.

A number of authors associate the anticoagulant activity of fucoidans with an inhibitory effect on thrombin (factor IIa) and factor Xa. This mechanism plays a leading role in the inhibition of thrombin formation by fucoidan [27,28,51].

**Figure 4:**
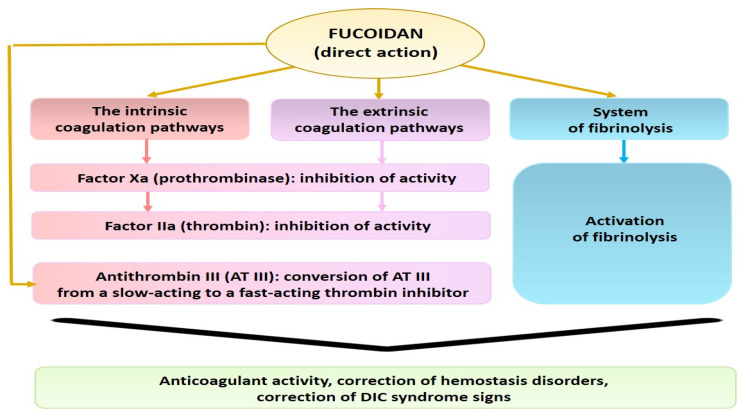
Scheme of the mechanisms of *F. evanescens* fucoidan action on the hemostasis system.

For example, it has been shown that fucoidan from the brown alga *Fucus evanescens* provides an inhibitory effect on thrombin (IIa) and factor Xa, both at the initial and subsequent stages of coagulation pathways. The authors believe that fucoidan, like heparin, converts AT III from a slow-acting to a fast-acting thrombin inhibitor [52,53] (Figure 4).

According to Jung et al. [54], fucose-containing sulfated PSs from *Ecklonia cava* strongly and selectively (factors VII, X, and II) enhanced ATIII-mediated coagulation-factor inhibition in both the extrinsic and common coagulation pathways [54]. According to Melo et al. [51], the anticoagulant activity of sulfated PSs is achieved mainly due to the potentiation of plasma cofactors, including AT III above. In particular, sulfated PSs (galactans) can bind to AT III above. However, the authors emphasize that the structural basis of the interactions is very complex due to the heterogeneity of these PSs. The interaction of sulfated galactans with the antithrombin/thrombin complex requires significantly longer chains than heparin, and these connections are made with other sites [51]. Becker et al. [55] emphasized that the nature of the specific interaction of sulfated PSs and galactans with AT III can determine their differences with heparin in the mechanisms of anticoagulant activity.

As presented above, the study of the anticoagulant effects of PSs was conducted using in vitro or in vivo experiments with parenteral administration. Polysaccharides are one of the most studied molecules as potential antithrombotic agents, and questions of oral bioavailability are important for the clinical use of PSs. As noted by Wang et al. [56], in future studies, a clear understanding of technical problems concerning the preparation, quality standards, and administration of fucoidan, including oral, must be defined to fully harness its therapeutic potential.

In this regard, numerous studies demonstrated the biological effects of sulfated PSs, including anticoagulant activity, with oral administration. For example, long-term (up to 6 months) oral administration of fucoidan from *Laminaria japonica* to Wistar rats at a dose of 300 mg/kg of body weight per day did not show significant side effects, but increasing the dose to 900 and 2500 mg/kg significantly prolonged the time of blood coagulation [57]. Fucoidans from *F. vesiculosus* and *L. japonica* (MW 10–300 kDa), administered subcutaneously or orally, did not cause negative changes in dogs with hemophilia A. Moreover, fucoidans led to normalization of hemostasis parameters [58]. Oral administration of fucoidan from *Cladosiphon okamuranus* to Wistar rats at a dose of 600 mg/kg per day did not cause significant changes in hemostasis parameters, while higher doses (1200 mg/kg) caused an increase the time of blood coagulation [59]. Zhao et al. [60] conducted a comparative study of the oral absorption, bioavailability, and antithrombotic activity of two fractions of fucoidan from *L. japonica* with low and medium MW on a model of electricity-induced arterial thrombosis in rats. The authors showed that a single administration of low-molecular-weight fucoidan at a dose of 400 and 800 mg/kg for 30 days inhibited the formation of arterial thrombosis, which was accompanied by moderate anticoagulant activity, significant antiplatelet activity, and effective fibrinolysis. The authors recommended this fucoidan as an oral antithrombotic agent, which is a highly desirable property of oral drugs [60].

## 3. Possible Pathophysiological Mechanisms Involved in the Hemostasis Disorders in the Pathological Progression of COVID-19

### 3.1. Systemic Endothelial Dysfunction

Hemostasis disorders in COVID-19 are largely determined by systemic endothelial dysfunction. Damage to the endothelium is caused by the fact that SARS-CoV-2 virus enters the human body and binds with a high avidity to the ACE2 (angiotensin-converting enzyme 2) receptor, present on endothelial cells and on the surface of type II alveolocytes [61]. This enzyme performs a protective function on endothelial cells and alveolocytes due to its anti-inflammatory, antithrombin, and antioxidant activities [62]. Direct evidence of endothelial-cell infection with the SARS-CoV-2 virus was found, leading to diffuse endothelial damage and death of the affected cells [61].

Systemic endothelial dysfunction and its activation serve as prerequisites for further pathophysiological events. Damaged or cytokine-activated endothelial cells, as well as monocytes, produce tissue factor (TF). TF plays a key role in the pathogenesis of hypercoagulation disorders in COVID-19. As a result, excessive formation of thrombin occurs, which leads to the formation of numerous thrombus in various organs, primarily leading to pulmonary thrombosis. This implication is called COVID-associated coagulopathy. According to some authors, thrombotic coagulopathy is present in almost three-quarters of COVID-19 patients admitted to the intensive care unit [2,63,64,65]. Due to the formation of microthrombi and impaired blood flow in the lungs, the death of lung-tissue cells occurs.

A significant contribution to the pathogenesis of hemostatic disorders and the development of thrombosis is made by hypoxemia resulting from damage to lung tissue [66,67,68].

In SARS-CoV-2 infection, hyperproduction of chemokines and proinflammatory cytokines (TNF, IL-1, IL-6, IL-8) and hyperactivation of immune cells, known as a cytokine storm, contribute to increased thrombin production and the likelihood of developing thrombosis, as well as the development of multiorgan failure (PON) in patients [3,63,69,70]. IL-6 is known to be a key activator of coagulopathy by inducing TF expression and increasing production of fibrinogen and platelets [71].

### 3.2. COVID-19-Associated Coagulopathy (CAC). Thrombotic Complications

Hypercoagulation abnormalities, which are detected at the initial stages of COVID-19, differ from the usual disseminated intravascular coagulation (DIC) syndrome, which develops in the later stages as the disease progresses, into the development of multi-organ failure, the addition of infectious complications, and sepsis [2,3,64].

Unlike the pattern seen in classic DIC syndrome, COVID-19-associated coagulopathy (CAC) characterized by minimal prolongation of coagulation-based laboratory parameters (activated partial thromboplastin time (APTT) and/or prothrombin time (PT)), or by a complete absence of changes in these parameters [64,72]. Mild thrombocytopenia (platelet count 100–150 × 10^9^/L) is also noted, while hypofibrinogenemia is rarely detected [73], or an increased level of fibrinogen is noted [72]. According to Yang et al. [74] and Liu et al. [75], thrombocytopenia has been observed in 18–36% of hospitalized patients with COVID-19, but is usually not severe.

A common complication of severe SARS-CoV-2 is venous (VTE) or arterial (ATE) thromboembolism, which is also the leading cause of death in this disease [5,63,76].

Venous thromboembolism (VTE) manifests as deep vein thrombosis (DVT) and pulmonary embolism (PE). As Klok et al. [5] noted, the most common thrombotic complication in COVID-19 patients was PE.

Arterial (ATE) thromboembolism is manifested by myocardial infarction, ischemic stroke, thrombosis and embolism of the peripheral arteries, and microvascular thrombotic disorders, which are often documented at autopsy [5,76]. According to Klok et al. [5], the incidence of thrombotic complications is 16–69% in patients with COVID-19 admitted to intensive care.

### 3.3. Thrombocytopenia: The Role of Platelets in Thromboembolism

Platelets play a key role in the development of arterial thromboembolism [77], and platelets are a potential target for the prevention of complications in COVID-19 [78].

In COVID-19 patients, the platelet count is often normal or mildly reduced, and according to N. Tang et al., thrombocytopenia occurs only in 12–36% of patients, and only in 5% of patients, the platelet counts are <100 × 10^9^/L [3]. Despite this, severe thrombocytopenia correlates with disease progression, as more than 55% of fatal COVID-19 patients have platelet counts of <100 × 10^9^/L. Thus, Yang et al. [74] noted that the intrahospital lethality was increased threefold in COVID-19 patients with thrombocytopenia.

Considering the mechanisms of thrombocytopenia in COVID-19 patients, the authors admit their multifactorial etiology, including cytokine storm, direct cytopathic effect of the virus on the bone marrow, and the formation of autoimmune complexes. This results in the destruction of platelets, inducing damage to the endothelium of pulmonary capillaries, causing platelet activation, aggregation, and reduction of circulating platelets [79,80]. The other authors [81] add that thrombocytopenia develops due to the fact, that viruses cause extensive damage to the bronchoalveolar tissue and the associated endothelial cells. This results in the intense platelet recruitment to the lungs and their consumption due to intense activation, which leads to depletion of the peripheral platelet count [81]. There are other mechanisms of thrombocytopenia in COVID-19, including the production of autoantibodies or immune complexes that mediate clearance and direct infection of hematopoietic progenitor cells and the megakaryocyte line, resulting in decreased platelet production [82].

Based on the above, it follows that monitoring the platelet count during hospitalization is very important for the prognosis in patients with COVID-19.

## 4. Treatment Strategies of COVID-19-Induced Hypercoagulation and the Potency of Seaweed Sulfated PSs for the Correction of Hemostasis Disorders

The treatment of COVID-19 patients, and in particular hemostasis disorders, is challenging and a difficult issue, since the pathogenesis of the disorders is not completely clear.

Addressing the need for the use of anticoagulants, their optimal dosage in the prevention and treatment of patients with COVID-19, and the control of anticoagulant therapy are important issues. Heparin drugs are often used as anticoagulants in the treatment of patients with COVID-19. There is unfractionated heparin (UFH)—first-generation preparations called heparin, which are a mixture of PSs with a molecular weight (MW) in the range of 2–30 kDa (with a predominance of high-molecular fractions of glycosaminoglycan), and low-molecular-weight heparin (LMWH), obtained by chemical or enzymatic hydrolysis of UFH and having a molecular weight of 3–7 kDa.

The mechanism of anticoagulant action of heparin is to inhibit the activity of thrombin (clotting factor IIa), which catalyzes the conversion of fibrinogen to fibrin and some other reactions in the hemostatic system. The antithrombin activity of heparin depends on the presence of the plasma protein antithrombin III (AT III). When heparin binds to AT III, conformational changes occur in the latter molecule, which allow it to quickly connect to the active center of thrombin and other serine proteases (factors IXa, Xa, XIa and XIIa, kallikrein, and plasmin) [83]. Therefore, heparin inhibits thrombosis, contributing to the inactivation of thrombin by its physiological inhibitor AT III. In the presence of heparin, the inactivation of thrombin by antithrombin III is accelerated about 1000-fold. Slightly less important for the manifestation of the anticoagulant effect of heparin is the “heparin II cofactor” (HC II), the second heparin-dependent thrombin inhibitor other than AT III, which neutralizes thrombin only at high concentrations of heparin in the blood plasma [84,85].

Both UFH and LMWH form complexes with AT III. However, if the UFH-AT III complex equally inhibits thrombin (factor IIa) and factor Xa, as well as other enzyme blood-clotting factors (Hageman factor, factors IX, XI, XII, etc.), then the anti-Xa activity prevails in LMWH [83,86,87,88].

Among the direct acting parenteral anticoagulants, it is recommended to give preference to LMWH compared to UFH, since the inhibitory activity of LMWH is stronger toward factor Xa, and LMWH rarely causes such complications as heparin—induced thrombocytopenia and osteoporosis. Qian et al. [89] observed a reduction of treatment duration in patients with acute exacerbation of chronic obstructive pulmonary disease receiving ventilatory support using LMWH. ISTH interim guidance recommends LMWH as a first-line therapy for the prophylaxis of VTE in hospitalized patients with COVID-19 [8]. The synthetic drug fondaparinux has also been shown to be effective for the prevention of VTE in older acute medical patients and in reducing total mortality [90].

According to Tang et al. [7], LMWH contributed to a decrease in mortality in patients with sepsis-induced coagulopathy. The same author has shown the effectiveness of enoxaparin for prophylactic coagulation abnormalities in COVID-19 patients.

With regard to the dosage of heparin, a prophylactic dose of LMWH is recommended for the prevention of VTE in hospitalized patients with COVID-19, and a therapeutic dose of LMWH is recommended for patients with significantly elevated D-dimer concentrations [91].

Therefore, taking into account the pathogenesis, prevention, and treatment regimens for severe complications of COVID-19, LMWH are included in the treatment protocols for all hospitalized patients. LMWH is also recommended for use in the outpatient setting. The administration of LMWH, the duration of its use, and the dose should be determined taking into account the risk factors for each patient in combination with laboratory monitoring.

Prospective clinical trials are ongoing to confirm the benefits of using anticoagulants to improve the survival of COVID-19-patients.

In addition, direct oral anticoagulants are used in COVID-19 patient therapy. However, as Testa et al. [92] have shown, when used concomitantly with antiviral agents, the latter, especially those that interact with P-glycoprotein and/or cytochrome P450-based metabolic pathways, can alter the pharmacokinetic and pharmacodynamic profiles, hence altering their anticoagulant activity and increasing the risk of bleeding. In this regard, although direct oral anticoagulants are convenient for the outpatient management of COVID-19 patients, caution is recommended, given their interaction with other drugs used to treat COVID-19 [93,94].

The use of heparins as anticoagulants is also indicated when taking into account the additional mechanisms of their action. In particular, heparin exhibits anti-inflammatory activity by inhibiting the recruitment of neutrophils into tissues, binding and neutralizing inflammatory cytokines and acute phase proteins, and potentially having a protective effect on the endothelium [95,96,97,98]. It also has been hypothesized that heparin may interfere with the interaction between the virus and the host cell via a nonspecific ion bond, and thus may contribute to reducing the frequency of infected cells in COVID-19 [96]. A number of studies have reported that the use of heparins in critically ill COVID-19 patients contributed to a decrease in the production of elevated cytokines (IL-6 and TNF-α) [99]. Additionally, heparin protects the endothelium [100]. There are also data that heparin interacts with spike proteins of several viruses, including the SARS-CoV-2 spike protein receptor-binding domain, suggesting that it may be able to modulate protein’s interactions with the endothelium [101]. Due to these characteristics, LMWH remains as the best choice of anticoagulant for hospitalized patients with severe COVID-19.

However, despite evidence that heparin in prophylactic doses is effective in reducing mortality in severe COVID-19 patients, a number of studies have reported a high incidence of venous and even arterial thromboembolic complications (deep vein thrombosis, pulmonary embolism or in situ thrombosis in the pulmonary arteries, and arterial thrombosis), despite thromboprophylaxis, which raises the issue of increasing anticoagulant doses. Prospective studies are currently in progress to try to answer this question [5,102,103].

White et al. [104] also reported heparin resistance in COVID-19. According to the authors, out of 14 patients with COVID-19 associated coagulopathy and with a high risk of thrombosis, who were treated with LMWH or UFH, resistance to UFH was documented in 8/10 (80%) patients, and suboptimal peak anti-Xa following therapeutic LMWH in 5/5 (100%) patients.

There is also the problem of anticoagulants’ optimal dose strategy. For example, a reduction in mortality in hospitalized patients with COVID-19 is shown when using a therapeutic, but not a preventive, dose of anticoagulants [12,13,105].

Taking into account the high frequency of thromboembolic complications, the International Society on Thrombosis and Haemostasis’ guidelines continue to recommend thromboprophylaxis for all hospitalized patients, and in most patients with COVID-19, thromboprophylaxis should be continued after hospital discharge [9].

The Expert Group of American Society of Hematology recommended the use of prophylactic-intensity anticoagulation compared to moderate-intensity or therapeutic-intensity anticoagulants for critical COVID-19-related patients or acute COVID-19 patients who do not have a confirmed VTE [106]. Taking into consideration their activation and their role in the clot formations, platelets are a potential target for the prevention of complications in SARS-CoV-2 infection [78,107,108,109]. The most commonly used antiplatelet agents are aspirin, clopidogrel, lopinavir/ritonavir, vorapaxar, and dipyridamole [109,110,111,112]. Among the antiplatelet agents, aspirin and clopidogrel are associated with decreased risk of ARDS, as well as decreased mortality among critically ill COVID-19 patients [111,112].

Vorapaxar and dipyridamole are considered promising antiplatelet agents for the treatment of COVID-19. Vorapaxar exhibits its antiplatelet activity through the antagonism of protease-activated receptor 1 (PAR-1), which plays an important role in thrombin-induced platelet aggregation, and is associated with blood clotting and inflammation [113].

For example, Liu et al. [110] used dipyridamole with a positive clinical effect. However, these authors note that there are a number of questions regarding the use of antiplatelet drugs in COVID-19 treatment, and therefore additional clinical trials are needed [110].

The data presented above indicate that anticoagulant and antithrombotic therapy play a key role in the treatment of hemostasis disorders in COVID-19, but there are still a number of unresolved issues regarding the use of these drugs. Thus, the optimal effective anticoagulant and antithrombotic agents, as well as their doses, remain uncertain.

A separate problem is the interaction of antithrombotic and anticoagulant drugs with specific drugs in COVID-19 treatments, since not all drugs are compatible. An individual approach to the patient is recommended, aimed at the optimal risk/benefit ratio of various antithrombotic strategies, taking into account the underlying hypercoagulable state.

Therefore, the treatment of patients and prevention of thrombosis in COVID-19 requires solving issues related to the need to find new effective anticoagulants, as well as their optimal dosage. In this regard, the search for new effective anticoagulants remains relevant. In this aspect, the sulfated PSs of seaweed are of interest.

Diagnostic criteria for assessing hemostasis are the following coagulation-based laboratory parameters: both basic tests, including PT with international normalized ratio (INR), APTT, TT, fibrinogen level, platelet count, and extended parameters—AT III and D-dimer level. So, the COVID-19-associated coagulopathy (CAC) markers are prolongation of APTT and PT, hypofibrinogenemia or hyperfibrinogenemia, thrombocytopenia, a decrease or increase in AT III level, progressive decrease in the fibrinogen level, and an increase in the concentration of D-dimer. An increase in the D-dimer level correlates with higher mortality, and rapidly increasing hypofibrinogenemia foreshadows the development of DIC [3,72,114,115]. In general, such parameters as D-dimer, PT, platelet count, and fibrinogen are important predictors of critical conditions, and are of particular prognostic significance in patients with COVID-19.

As follows from our review, the major therapeutic role of seaweed sulfated PSs, as well as heparin, is in the inhibition of clot formation. Due to anticoagulant, antithrombotic, and fibrinolytic activities, it is possible to use PSs both for the purposes of thrombosis prevention and for thrombolysis. The mechanisms of anticoagulant and antithrombotic activity of seaweed sulfated PSs include the impact on both the factors of the extrinsic and intrinsic coagulation pathways, and the final stage of coagulation—the conversion of fibrinogen to fibrin under the thrombin influence. Anticoagulant activity of PS can be associated with plasma AT III, and antithrombotic activity—with an increase in the level of t-PA and competitive binding to the t-PA-PAI-1 complex. In this regard, seaweed sulfated PSs can contribute to normalization of the coagulation-based laboratory parameters (APTT, PT, AT III, D-dimer, and fibrinogen level); i.e., correction of hemostasis disorders in COVID-19 patients.

The use of seaweed sulfated PSs, as well as heparin drugs, for the prevention and treatment of thrombosis is promising both at the stage of hospitalization of patients, and after discharge from the hospital. PSs can also be recommended for use in the outpatient setting for a long time.

In addition to being strong anticoagulants, antithrombotics, and fibrinolytics, PSs are also able to exhibit other effects that are useful in the treatment of viral infections, accounting for known data on their antiviral activity [116,117,118,119], anti-inflammatory activity, and ability to reduce the proinflammatory cytokine production and other beneficial effects [17,56,120,121,122,123,124]. Such a wide range of biological activity is due to the chemical nature of PSs. As is known, seaweed sulfated PSs, as well as heparins, are mimetics of glycosaminoglycans (GAGs). In mammalians, GAGs in the composition of proteoglycans are constituents of connective tissue. The polyanionic nature and the ability to interact with proteins with different affinities are properties of GAGs that determine their biological function and their role as a potential agents in various applications. So, GAGs such as heparin or seaweed sulfated polysaccharides are capable of interacting with factors of the coagulation cascade during clotting-inhibition processes [125]. GAGs also are exploited by numerous microorganisms for cellular attachment, adhesion, and invasion and evasion of the host’s immune system. As for SARS-CoV and other coronaviruses, they can bind to host cells by clinging through their GAG [125]. In this regard, numerous works are devoted to the anticoronavirus ability of PSs [126,127,128].

## 5. Conclusions

Seaweed sulfated polysaccharides (PS) possess anticoagulant, antithrombotic, and fibrinolytic properties. These activities depends on the structural features (content and position of sulfate groups on the main chain of the PSs, molecular weight, monosaccharide composition and type of glycosidic bonds, the degree of PS chain branching, etc.). Sulfated PSs act on the hemostasis system both directly and indirectly and affect the factors of the extrinsic and intrinsic coagulation pathways, as well as the final stage of coagulation or the conversion of fibrinogen to fibrin under the thrombin influence. Anticoagulant activity of PS can be associated with plasma AT III, and antithrombotic activity—with an increase in the level of t-PA and competitive binding to the t-PA-PAI-1 complex.

The combination of the anticoagulant, antithrombotic, and fibrinolytic properties allows us to consider seaweed sulfated PSs as alternative sources of new anticoagulant and antithrombotic compounds. In this regard, and along with the low toxicity and relative low cost of their production, sulfated PSs are promising for clinical use and represent an alternative to heparin. The wide therapeutic potential of seaweed sulfated PSs, including anticoagulant, thrombolytic, and fibrinolytic activities, opens up new possibilities for their study in experimental and clinical trials. These natural compounds can be important complementary drugs in the fight against coronaviruses due to their natural origin, safety, and low cost compared to synthetic drugs. However, further investigations are needed regarding the use of seaweed PSs as potential anticoagulants, thrombolytics, and fibrinolytics aimed to correct hemostasis disorders in COVID-19 patients.
